# Predicting End-Stage Renal Disease and Mortality in Chronic Kidney Disease Using Machine Learning: Retrospective Cohort Study

**DOI:** 10.2196/81152

**Published:** 2026-06-05

**Authors:** Tz-Heng Chen, Kuan-Hsun Lin, Yang Ho, Wei-Cheng Tseng, Yuan-Chia Chu, Der-Cherng Tarng

**Affiliations:** 1 Division of Nephrology, Department of Medicine, Taipei Veterans General Hospital, No 201, Sec 2, Shipai Rd, Beitou District, Taipei, 11217, Taiwan; 2 School of Medicine, College of Medicine, National Yang Ming Chiao Tung University, Taipei, Taiwan; 3 Institute of Emergency and Critical Care Medicine, National Yang Ming Chiao Tung University, Taipei, Taiwan; 4 Department of Information Management, Taipei Veterans General Hospital, No 201, Sec 2, Shipai Rd, Beitou District, Taipei, 11217, Taiwan, +886 986-680623, 886 2-28757841; 5 Department of Information Management, National Taipei University of Nursing and Health Science, Taipei, Taiwan; 6 Big Data Center, Taipei Veterans General Hospital, Taipei, Taiwan; 7 Department of Medicine, Taipei Veterans General Hospital, Taipei, Taiwan

**Keywords:** chronic kidney disease, end-stage renal disease, machine learning, mortality, Shapley additive explanations, SHAP

## Abstract

**Background:**

Chronic kidney disease (CKD) is a global health burden characterized by heterogeneous progression trajectories. Without timely and appropriate management, CKD can lead to increased morbidity and mortality and a reduced quality of life. Therefore, early identification of patients at high risk of developing end-stage renal disease (ESRD) or mortality is essential to facilitate timely intervention and improve patient outcomes.

**Objective:**

This study aimed to develop and validate machine learning models to predict ESRD and all-cause mortality in patients with CKD.

**Methods:**

We developed and validated machine learning models using data from patients with CKD and an estimated glomerular filtration rate of <60 mL/min/1.73 m^2^, who were treated at Taipei Veterans General Hospital between 2011 and 2021. Predictors included 69 routinely available demographic, clinical, medication, laboratory, and echocardiographic variables. The outcomes were ESRD and all-cause mortality. The cohort was randomly divided into training (n=23,741, 80%) and testing (n=5936, 20%) sets. The evaluated models included extreme gradient boosting, light gradient boosting machine, categorical boosting, random forest, and a stacking classifier. Model performance was assessed using the area under the receiver operating characteristic curve (AUROC), the area under the precision-recall curve, calibration, and decision-curve analysis. Supplementary time-to-event analyses were performed using the kidney failure risk equation and survival-based machine learning models.

**Results:**

A total of 29,677 patients were included in the study. The median age was 79 (IQR 70.0‐88.0) years, and 16,359 (55.1%) were male. Among these patients, 14,993 (50.5%) had hypertension, 7908 (26.6%) had diabetes mellitus, and 1768 (6%) had cancer. During follow-up, 649 patients (2.2%) developed ESRD and 3631 (12.2%) died. The models demonstrated high predictive performance for ESRD, with AUROCs ranging from 0.839 to 0.894. For all-cause mortality, the predictive performance was more modest, with AUROCs ranging from 0.752 to 0.774. Given the low incidence of ESRD in this cohort, model performance was additionally evaluated using precision-recall curves. The area under the precision-recall curve ranged from 0.172 to 0.216 for ESRD prediction and from 0.330 to 0.356 for all-cause mortality across models. Calibration and decision-curve analyses supported model reliability and clinical utility.

**Conclusions:**

Machine learning algorithms may serve as useful tools for risk stratification of ESRD and all-cause mortality in patients with CKD, with the potential to support more individualized clinical management.

## Introduction

Chronic kidney disease (CKD) is recognized as a major global public health challenge, with substantial impacts on morbidity and health care utilization worldwide [1,2]. In Taiwan, CKD affects approximately 10% of the adult population. The consistently high end-stage renal disease (ESRD) incidence imposes a substantial burden on the health care system and is accompanied by steadily increasing financial pressure [3,4]. This local epidemiologic context supports the need to improve risk stratification for kidney failure and mortality using Taiwan-based real-world data. Despite advancements in CKD research and management, accurately predicting progression to ESRD remains challenging. CKD is heterogeneous, with diverse etiologies, clinical presentations, and pathological features, and progression rates can vary substantially even among patients with identical primary kidney diseases [5]. Numerous clinical factors, including baseline estimated glomerular filtration rate (eGFR), proteinuria levels, age, and comorbidities, have been identified as contributors to CKD progression [6,7]. However, these factors and traditional risk equations, such as the kidney failure risk equation (KFRE), may not fully capture the complex, multidimensional nature of CKD, in which clinical, laboratory, and demographic variables can interact nonlinearly.

In recent years, advancements in artificial intelligence have significantly impacted the medical field. The integration of machine learning and deep learning with big data has found extensive applications across various medical disciplines, including image interpretation, disease diagnosis, and risk prediction [8]. Previous research has highlighted the potential of artificial intelligence for predicting the risk of patients with diabetes developing diabetic kidney disease or progressing to ESRD [9,10]. By incorporating a comprehensive range of clinical, demographic, and laboratory variables, these machine learning models have demonstrated considerable promise for achieving more precise risk stratification compared to traditional methods. Despite these advantages, several challenges remain in translating machine learning models into clinical practice. Many models have limited transparency, which can reduce interpretability and clinician trust [11]. In addition, differences in data sources, preprocessing strategies, and modeling pipelines may affect reproducibility across studies [12,13]. Furthermore, a substantial proportion of published machine learning prediction models lack external validation, which raises concerns about their generalizability and real-world clinical usefulness [14]. To bridge this gap between predictive modeling and clinical application, there is a critical need for transparent algorithms that can accurately handle the complex, time-to-event nature of chronic disease progression. Integrating tools such as Shapley additive explanations (SHAP) and survival-based machine learning techniques can demystify these models, providing clinicians with actionable insights into individual risk trajectories over time.

The aim of this study was to develop and validate machine learning–based models to predict the risks of ESRD and all-cause mortality in patients with CKD using a large-scale single-center cohort. We hypothesized that these models would enhance the precision of risk stratification, identify clinically meaningful predictors to inform clinical decision-making, and ultimately facilitate timely interventions to improve patient outcomes.

## Methods

### Data Sources and Study Population

This retrospective cohort study involved patients with CKD, defined as persistent eGFR <60 mL/min/1.73 m² for at least 3 months. Data were comprehensively obtained from the Taipei Veterans General Hospital Big Data Center, covering the period from January 2011 to December 2021. Patient information included demographic data, comorbidities, medication prescriptions, laboratory results, and echocardiography findings. Exclusion criteria were prior renal replacement therapy (dialysis or kidney transplantation) before study enrollment and individuals younger than 20 years at the time of CKD diagnosis. eGFR calculations used the 2021 Chronic Kidney Disease Epidemiology Collaboration equation [15].

### Feature Selection

Our machine learning models incorporated 69 features across multiple clinical domains, comprising a broad set of routinely available electronic health record (EHR) variables to capture multidimensional risk domains relevant to CKD progression and all-cause mortality. Demographic and lifestyle characteristics included age, sex, body weight, BMI, systolic and diastolic blood pressure, alcohol consumption, betel nut chewing, and cigarette smoking. Comorbidity profiles included hypertension, diabetes mellitus, and cancer. Medication history encompassed antidiabetic agents (metformin, sodium-glucose cotransporter 2 inhibitors, glucagon-like peptide-1 receptor agonists, sulfonylureas, α-glucosidase inhibitors, thiazolidinediones, dipeptidyl peptidase-4 inhibitors, insulin), cardiovascular drugs (statins, aspirin, clopidogrel, direct oral anticoagulants, warfarin, angiotensin-converting enzyme inhibitors, angiotensin II receptor blockers, beta-blockers, calcium channel blockers, thiazide diuretics, loop diuretics, mineralocorticoid receptor antagonists, nitrates, sacubitril or valsartan), and anti-inflammatory medications (nonsteroidal anti-inflammatory drugs, cyclooxygenase-2 inhibitors). Laboratory parameters included serum creatinine, blood urea nitrogen, sodium, potassium, chloride, bicarbonate, uric acid, calcium, phosphate, serum albumin, total protein, creatine kinase, glucose, hemoglobin A_1c_, total cholesterol, high-density lipoprotein cholesterol, low-density lipoprotein cholesterol, triglycerides, alanine aminotransferase, alkaline phosphatase, gamma-glutamyl transferase, total bilirubin, international normalized ratio, free thyroxine, thyroid-stimulating hormone, white blood cell count, hemoglobin, platelet count, urine protein-to-creatinine ratio (UPCR), and urine albumin-to-creatinine ratio (UACR). Cardiac function was evaluated using left ventricular ejection fraction via echocardiography.

### Outcome Definition

The primary clinical outcomes were progression to ESRD and all-cause mortality. Both outcomes were ascertained using electronic health records from the Taipei Veterans General Hospital. To prevent the misclassification of temporary acute dialysis, ESRD was strictly defined as the presence of dialysis-related procedure codes recorded for at least 3 consecutive months, or the occurrence of kidney transplantation identified by the *International Classification of Diseases, Ninth or Tenth Revision*, Clinical Modification codes. Outcomes were represented as binary variables, with 1 indicating occurrence and 0 indicating nonoccurrence during follow-up.

### Data Preprocessing

Categorical variables were summarized as frequencies (percentages) and continuous variables as medians with IQRs. The dataset was randomly divided into training (23,741/29,677, 80%) and testing (5936/29,677, 20%) sets using stratified sampling with a fixed random seed to preserve the outcome distribution. Infinite values were converted to missing values, and only numeric variables were retained for model development. In the primary analysis, missing values were imputed using K-nearest-neighbor imputation (*k*=5), fitted on the training set, and then applied to the testing set. Continuous variables were standardized using a StandardScaler fitted on the training set. To address significant class imbalance for ESRD, the Synthetic Minority Over-sampling Technique (SMOTE) was applied to the training data only with a sampling strategy of 0.9.

### Machine Learning Models

The analysis used five advanced machine learning models: extreme gradient boosting (XGBoost), light gradient boosting machine (LightGBM), categorical boosting (CatBoost), random forest, and a stacking classifier integrating these base models. Each tree-based algorithm applied ensemble learning with distinct optimization strategies. The stacking classifier used logistic regression as a meta-learner to leverage complementary strengths, reduce variance, and enhance overall predictive accuracy.

### Hyperparameter Optimization

Hyperparameter optimization was conducted using randomized search combined with 3-fold cross-validation, targeting the maximization of the area under the precision-recall curve (AUPRC), which is more appropriate for imbalanced outcomes. The optimization explored 30 random combinations within predefined parameter ranges, identifying optimal configurations efficiently. Key hyperparameters included learning rates, tree depths, regularization parameters, and specific configurations for each algorithm. The stacking classifier used logistic regression as the final meta-learner.

### Model Evaluation and Feature Importance

Model performance was comprehensively evaluated using multiple metrics, with the area under the receiver operating characteristic curve (AUROC) as the primary measure of discrimination. Additional evaluation metrics included the AUPRC, accuracy, precision, recall, *F*_1_ score, specificity, and log loss. Confusion matrices were also generated using the optimal cutoff determined by the Youden index. Calibration and clinical utility were further assessed using calibration curves and decision-curve analysis. Feature importance was evaluated using SHAP, which quantified the contribution of individual variables to model predictions and facilitated interpretation of clinically relevant predictors.

### Survival Analyses

As time-to-event analyses for ESRD, we evaluated the KFRE and random survival forest (RSF) at 2-year and 5-year horizons. To ensure a fair comparison, these models were evaluated using a unified horizon-specific test mask requiring landmark eligibility, complete age, sex, eGFR, and UACR inputs and nonmissing model predictions. Time-dependent discrimination was assessed using inverse probability of censoring weighting–based AUROC.

### Sensitivity Analyses

Sensitivity analyses were performed to assess the robustness of model performance. First, an age-stratified analysis was conducted using the XGBoost model to evaluate discrimination separately in patients aged ≥60 years and <60 years in the testing set. Second, to examine the influence of missing-data handling, we repeated model evaluation without imputation using complete-case data only. Model discrimination in these sensitivity analyses was assessed using AUROC.

### Implementation Details

Analyses were implemented using Python 3.9 with specialized libraries. Scikit-learn provided core machine learning functionality, supplemented by XGBoost, LightGBM, and CatBoost for gradient boosting. The stacking classifier was implemented using sklearn.ensemble.StackingClassifier with logistic regression as the final estimator. Pandas and NumPy facilitated data manipulation, while visualization was achieved using Matplotlib and Seaborn. Missing value imputation used sklearn.impute.KNNImputer, dataset partitioning used sklearn.model_selection.train_test_split, and class balancing used imblearn.over_sampling.SMOTE. Hyperparameter optimization applied sklearn.model_selection.RandomizedSearchCV, and stratified k-fold cross-validation used sklearn.model_selection.StratifiedKFold. Complementary statistical analyses were performed with SAS version 9.4 (SAS Institute, Cary, NC), and statistical significance was defined as *P*<.05. Complementary survival analyses were implemented using scikit-survival and PyTorch for time-to-event modeling and inverse probability of censoring weighting–based evaluation.

### Ethical Considerations

This study protocol was approved by the institutional review board of Taipei Veterans General Hospital (approval number 2023-07-001CC), with informed consent waived due to the use of deidentified data.

## Results

### Characteristics and Distribution of Patients

A total of 29,677 patients with CKD were enrolled during the 10-year study period. Detailed patient demographic data are presented in Table 1. The median patient age was 79.0 (IQR 70.0‐88.0) years, and 16,359 (55.1%) patients were male. Additionally, 14,993 (50.5%) patients had hypertension, 7908 (26.6%) patients had diabetes mellitus, and 1768 (6%) patients had cancer. Within the entire CKD cohort, 649 (2.2%) patients progressed to ESRD, including 519 of 23,741 (2.2%) in the training set and 130 of 5936 (2.2%) in the testing set. All-cause mortality occurred in 3631 of 29,677 patients (12.2%), including 2905 of 23,741 (12.2%) in the training set and 726 of 5936 (12.2%) in the testing set.

**Table 1.** Baseline demographic and clinical characteristics of the study cohort.

Characteristics	Full cohort (N=29,677)	Training set (n=23,741)	Testing set (n=5936)
Demographic data			
Age (y), median (IQR)	79.0 (70.0-88.0)	79.0 (70.0-88.0)	79.0 (70.0-88.0)
Male gender, n (%)	16,359 (55.1)	13,092 (55.2)	3267 (55.0)
Alcohol, n (%)	2990 (10.1)	2370 (10.0)	620 (10.5)
Betel nut, n (%)	608 (2.1)	487 (2.1)	121 (2.1)
Smoking, n (%)	3960 (13.4)	3158 (13.4)	802 (13.6)
BW^a^ (kg), median (IQR)	62.5 (54.0-71.8)	62.5 (54.0-71.7)	62.6 (54.0-72.2)
BMI (kg/m²), median (IQR)	24.5 (21.9-27.4)	24.5 (21.9-27.4)	24.7 (21.9-27.6)
SBP^b^ (mm Hg), median (IQR)	133.9 (122.0-146.9)	134.0 (122.0-146.8)	133.8 (122-147)
DBP^c^ (mm Hg), median (IQR)	72.1 (65-80)	72.0 (65-80)	72.2 (65-80)
Comorbidities, n (%)			
Hypertension	14,993 (50.5)	11,968 (50.4)	3025 (51.0)
Diabetes mellitus	7908 (26.6)	6335 (26.7)	1573 (26.5)
Cancer	1768 (6.0)	1422 (6.0)	346 (5.8)
Laboratory data, median (IQR)			
Creatinine (mg/dL)	1.2 (1.0-1.5)	1.2 (1.0-1.5)	1.2 (1.0-1.5)
BUN^d^ (mg/dL)	22.0 (18.0-29.0)	22.0 (18.0-29.0)	22.0 (17.0-29.0)
Sodium (mmol/L)	140 (138.0-142.0)	140 (138.0-142.0)	140 (138.0-142.0)
Potassium (mmol/L)	4.2 (3.9-4.5)	4.2 (3.9-4.5)	4.2 (3.9-4.5)
Chloride (mmol/L)	105.0 (101.0-107.0)	105.0 (101.0-107.0)	104.0 (101.0-107.0)
Uric acid (mg/dL)	6.4 (5.2-7.7)	6.4 (5.2-7.7)	6.5 (5.3-7.7)
Calcium (mg/dL)	9.1 (8.7-9.5)	9.1 (8.7-9.5)	9.1 (8.7-9.5)
Phosphate (mg/dL)	3.5 (3.1-4.0)	3.6 (3.1-4.0)	3.5 (3.1-4.0)
Albumin (g/dL)	3.9 (3.5-4.3)	3.9 (3.5-4.3)	3.9 (3.5-4.2)
Cholesterol (mg/dL)	167.0 (142.0-195.0)	167.0 (141.0-194.0)	168.0 (143.0-195.0)
Bicarbonate (mmol/L)	23.4 (20.2-26.7)	23.4 (20.2-26.6)	23.6 (20.3-27.0)
HbA_1c_^e^ (%)	6.3 (5.8-7.3)	6.4 (5.8-7.3)	6.3 (5.7-7.2)
HDL-C^f^ (mg/dL)	43.0 (36.0-53.0)	44.0 (36.0-53.0)	43.0 (36.0-52.0)
LDL-C^g^ (mg/dL)	95.0 (74.8-119.0)	95.0 (74.0-118.0)	96.0 (76.0-120.0)
Triglyceride (mg/dL)	114.0 (83.0-163.0)	114.0 (83.0-162.0)	117.0 (85.0-165.0)
Total protein (g/dL)	6.5 (5.9-7.2)	6.5 (5.9-7.2)	6.5 (5.8-7.1)
CK^h^ (U/L)	61.0 (37.0-114.0)	61.0 (37.0-112.0)	63.0 (37.0-120.0)
Glucose (mg/dL)	128.0 (106.0-168.0)	128.0 (106.0-168.0)	128.0 (106.0-168.0)
ALT^i^ (U/L)	19.0 (14.0-30.0)	19.0 (14.0-30.0)	19.0 (14.0-31.0)
ALKP^j^ (U/L)	76.0 (60.0-100.0)	76.0 (60.0-100.0)	75.0 (60.0-100.0)
GGT^k^ (U/L)	40.0 (21.0-92.0)	40.0 (21.0-92.0)	39.0 (21.0-91.0)
Total bilirubin (mg/dL)	0.5 (0.3-0.8)	0.5 (0.3-0.8)	0.5 (0.3-0.8)
INR^l^	1.0 (1.0-1.1)	1.0 (1.0-1.1)	1.0 (1.0-1.1)
Free T4^m^ (ng/dL)	1.1 (0.9-1.2)	1.1 (0.9-1.2)	1.1 (0.9-1.2)
TSH^n^ (µIU/mL)	1.5 (0.8-2.6)	1.5 (0.8-2.6)	1.5 (0.8-2.5)
WBC^o^ (per µL)	7100 (5600-9400)	7100 (5600-9400)	7160 (5600-9400)
Hemoglobin (g/dL)	12.3 (10.8-13.7)	12.3 (10.8-13.7)	12.4 (10.8-13.7)
PLT^p^ (per µL)	208,000 (164,000-259,000)	208,000 (164,000-259,000)	208,000 (164,000-258,000)
UPCR^q^ (g/g)	0.5 (0.1-2.2)	0.5 (0.1-2.2)	0.5 (0.2-2.3)
UACR^r^ (g/g)	0.1 (0.0-0.4)	0.1 (0.0-0.4)	0.0 (0.0-0.3)
Cardiac function, median (IQR)			
LVEF^s^ (%)	58.0 (53.0-63.0)	58.0 (53.0-63.0)	58.0 (53.0-62.0)
Concomitant medications, n (%)			
Metformin	5038 (17.0)	4056 (17.1)	982 (16.5)
SGLT2^t^ inhibitors	801 (2.7)	647 (2.7)	154 (2.6)
GLP1 RAs^u^	114 (0.4)	91 (0.4)	23 (0.4)
Sulfonylureas	2493 (8.4)	1986 (8.4)	507 (8.5)
Alpha-glucosidase inhibitors	889 (3.0)	724 (3.0)	165 (2.8)
Thiazolidinediones	492 (1.7)	395 (1.7)	97 (1.6)
DPP4^v^ inhibitors	1196 (4.0)	971 (4.1)	225 (3.8)
Insulin	3968 (13.4)	3194 (13.5)	774 (13)
Statins	7557 (25.5)	6023 (25.4)	1534 (25.8)
Aspirin	5538 (18.7)	4406 (18.6)	1132 (19.1)
Clopidogrel	3033 (10.2)	2414 (10.2)	619 (10.4)
DOACs^w^	1739 (5.9)	1396 (5.9)	343 (5.8)
Warfarin	674 (2.3)	530 (2.2)	144 (2.4)
Alpha blockers	2188 (7.4)	1731 (7.3)	457 (7.7)
Beta blockers	8378 (28.2)	6640 (28.0)	1738 (29.3)
CCB^x^	10,652 (35.9)	8497 (35.8)	2155 (36.3)
ACE^y^ inhibitors	856 (2.9)	664 (2.8)	192 (3.2)
ARB^z^	9699 (32.7)	7762 (32.7)	1937 (32.6)
Thiazides	902 (3.0)	724 (3.0)	178 (3.0)
Loop diuretics	5417 (18.3)	4312 (18.2)	1105 (18.6)
MRAs^aa^	2976 (10.0)	2382 (10.0)	594 (10.0)
Nitrates	3406 (11.5)	2680 (11.3)	726 (12.2)
NSAIDs^ab^	2490 (8.4)	1978 (8.3)	512 (8.6)
COX-2^ac^ inhibitors	3569 (12.0)	2850 (12.0)	719 (12.1)
Sacubitril or valsartan	297 (1.0)	231 (1.0)	66 (1.1)

a BW: body weight.

b SBP: systolic blood pressure.

c DBP: diastolic blood pressure.

d BUN: blood urea nitrogen.

e HbA_1c_: glycated hemoglobin.

f HDL-C: high-density lipoprotein cholesterol.

g LDL-C: low-density lipoprotein cholesterol.

h CK: creatine kinase.

i ALT: alanine aminotransferase.

j ALKP: alkaline phosphatase.

k GGT: gamma-glutamyl transferase.

l INR: international normalized ratio.

m Free T4: free thyroxine.

n TSH: thyroid-stimulating hormone.

o WBC: white blood cell count.

p PLT: platelet count.

q UPCR: urine protein-to-creatinine ratio.

r UACR: urine albumin-to-creatinine ratio.

s LVEF: left ventricular ejection fraction.

t SGLT2: sodium-glucose cotransporter 2.

u GLP1 RA: glucagon-like peptide-1 receptor agonist.

v DPP4: dipeptidyl peptidase-4.

w DOAC: direct oral anticoagulant.

x CCB: calcium channel blocker.

y ACE: angiotensin-converting enzyme.

z ARB: angiotensin II receptor blocker.

aa MRA: mineralocorticoid receptor antagonist.

ab NSAID: nonsteroidal anti-inflammatory drug.

ac COX-2: cyclooxygenase-2.

### Model Performance

**Figure 1.**
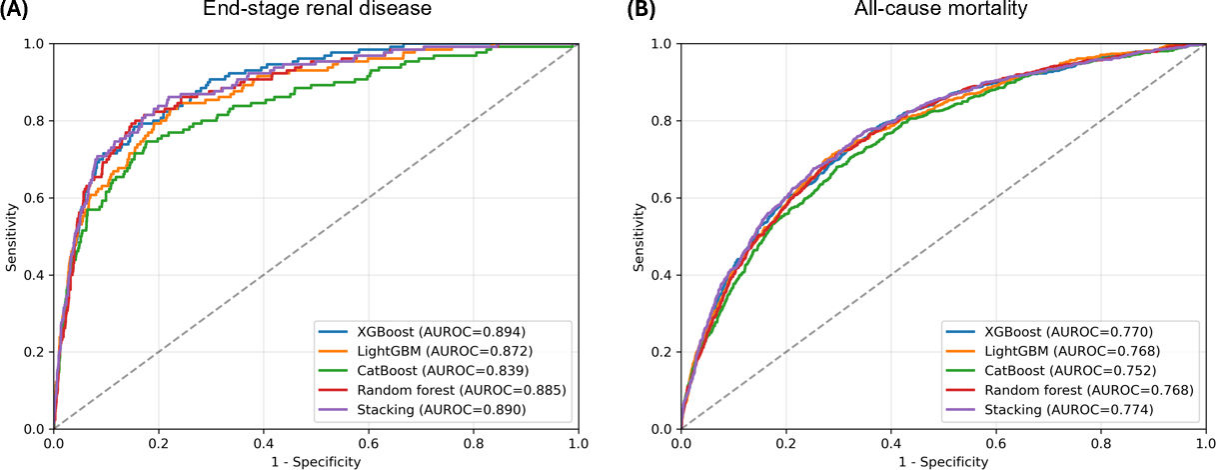
Receiver operating characteristic curves of the machine learning models for prediction of (A) end-stage renal disease and (B) all-cause mortality in the testing set. AUROC: area under the receiver operating characteristic curve; CatBoost: categorical boosting; LightGBM: light gradient boosting machine; XGBoost: extreme gradient boosting.

Five machine learning algorithms, including XGBoost, LightGBM, CatBoost, random forest, and a stacking classifier, were trained on 80% (n=23,741) of the cohort and evaluated on the remaining 20% (n=5936). Figure 1A displays ROC curves illustrating the predictive performance of each algorithm for ESRD prediction in the testing set. Overall, all models showed good discrimination, with AUROC values ranging from 0.839 to 0.894. XGBoost achieved the highest AUROC of 0.894, followed by the stacking classifier (0.890), random forest (0.885), LightGBM (0.872), and CatBoost (0.839).

Predictive performance for all-cause mortality was lower than that for ESRD, with AUROC values ranging from 0.752 to 0.774 (Figure 1B). The stacking classifier achieved the highest AUROC of 0.774, followed by XGBoost (0.770), LightGBM (0.768), random forest (0.768), and CatBoost (0.752).

**Figure 2.**
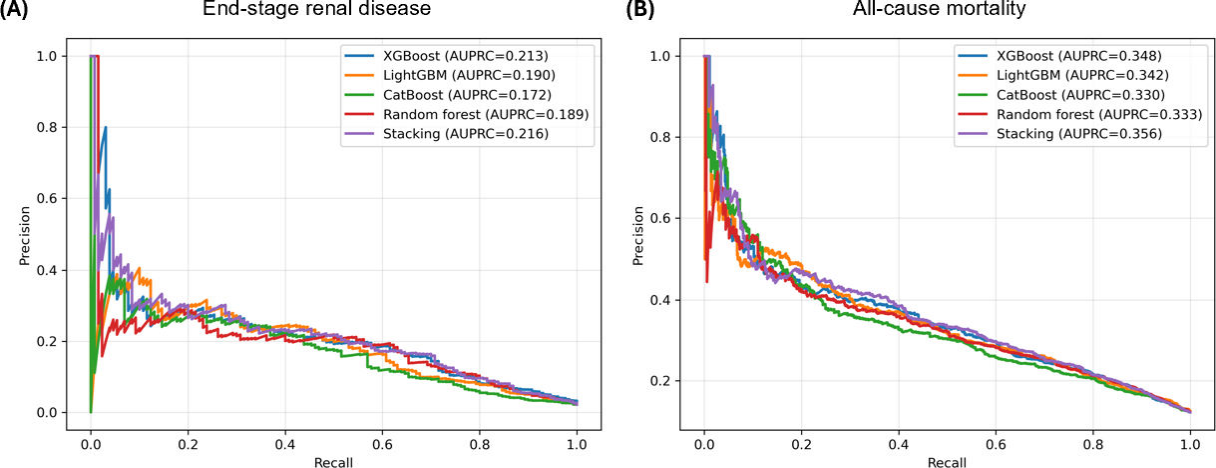
Precision-recall curves of the machine learning models for prediction of (A) end-stage renal disease and (B) all-cause mortality in the testing set. AUPRC: area under the precision-recall curve; CatBoost: categorical boosting; LightGBM: light gradient boosting machine; XGBoost: extreme gradient boosting.

Given the low incidence of ESRD in the study cohort, precision-recall analysis was additionally performed. The AUPRC ranged from 0.172 to 0.216 (Figure 2A). The stacking classifier achieved the highest AUPRC of 0.216, followed by XGBoost (0.213), LightGBM (0.190), random forest (0.189), and CatBoost (0.172). For all-cause mortality, the AUPRC ranged from 0.330 to 0.356 (Figure 2B). The stacking classifier achieved the highest AUPRC of 0.356, followed by XGBoost (0.348), LightGBM (0.342), random forest (0.333), and CatBoost (0.330). Additional performance metrics, including accuracy, precision, recall, *F*_1_ score, specificity, and log loss, are summarized in Tables S1 and S2 in Multimedia Appendix 1. Confusion matrices for ESRD and all-cause mortality prediction using the XGBoost model are shown in Figure S1 in Multimedia Appendix 1.

### Ranks of Feature Importance and SHAP Values in the XGBoost Model

**Figure 3.**
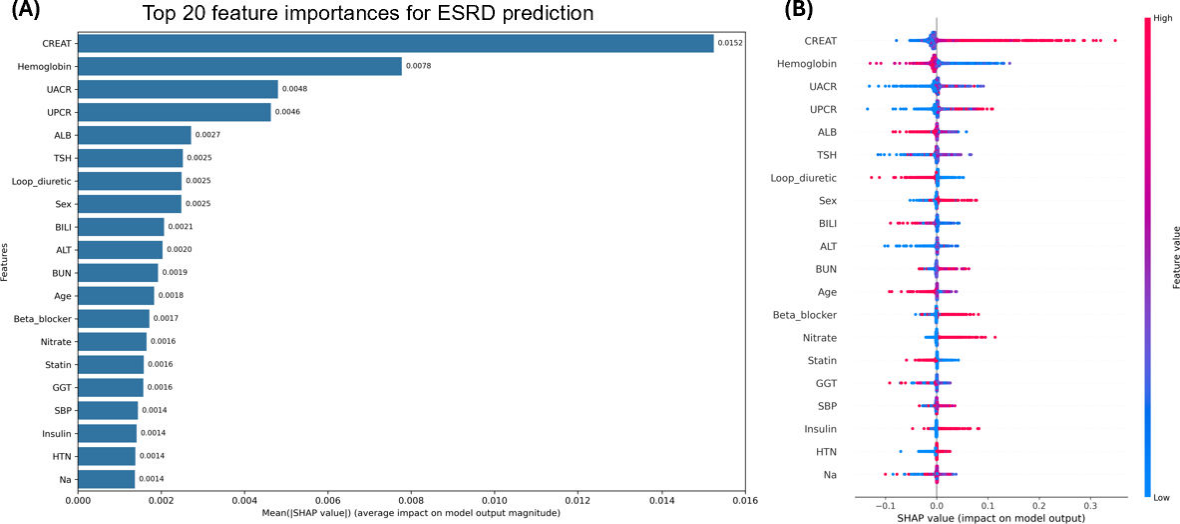
Shapley additive explanations (SHAP) analysis for the prediction of end-stage renal disease (ESRD). (A) Mean absolute SHAP values showing the top 20 most influential features in the extreme gradient boosting (XGBoost) model. (B) SHAP summary plot showing the direction and magnitude of feature effects on the model output. Red indicates higher feature values, and blue indicates lower feature values. ALB: albumin; ALT: alanine aminotransferase; BILI: total bilirubin; BUN: blood urea nitrogen; CREAT: creatinine; CI: chloride; GGT: gamma-glutamyl transferase; HTN: hypertension; Na: sodium; SBP: systolic blood pressure; TSH: thyroid-stimulating hormone; UACR: urine albumin-to-creatinine ratio; UPCR: urine protein-to-creatinine ratio.

Feature attribution analyses using SHAP identified key predictors for ESRD and all-cause mortality. Figure 3A presents feature importance rankings from the XGBoost model for ESRD prediction, arranged in descending order based on impact. The top 5 most influential features were serum creatinine, hemoglobin, UACR, UPCR, and albumin. Figure 3B illustrates the SHAP summary plot, where red points represent higher feature values and blue points indicate lower values. Positive SHAP values suggest increased ESRD risk, while negative values indicate lower risk. Higher values of serum creatinine, UACR, and UPCR, as well as lower hemoglobin and albumin levels, were associated with a higher predicted risk of ESRD.

**Figure 4.**
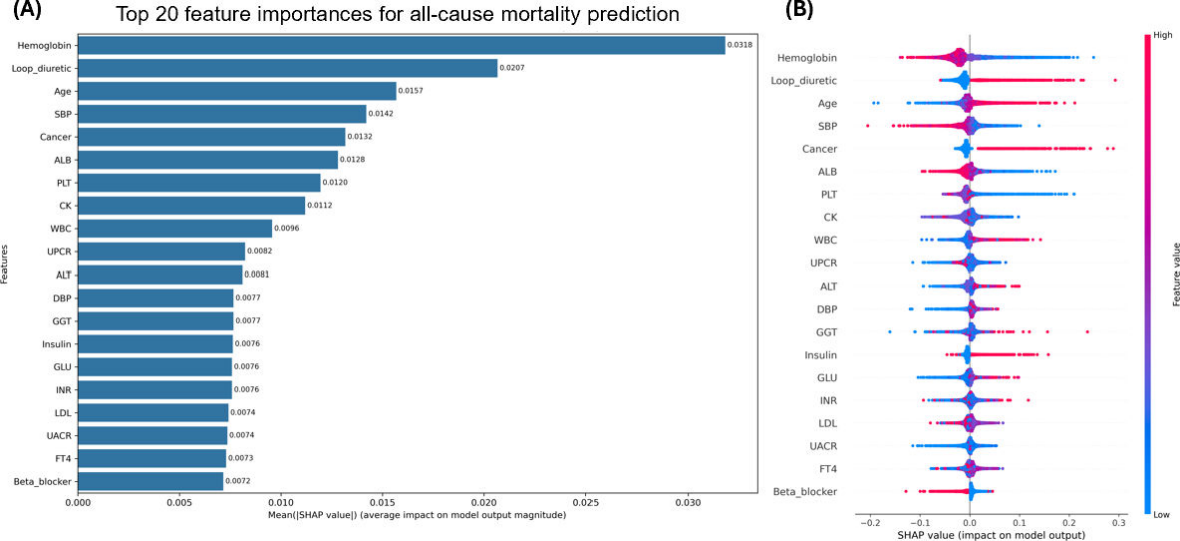
Shapley additive explanations (SHAP) analysis for the prediction of all-cause mortality. (A) Mean absolute SHAP values showing the top 20 most influential features in the extreme gradient boosting (XGBoost) model. (B) SHAP summary plot showing the direction and magnitude of feature effects on the model output. Red indicates higher feature values, and blue indicates lower feature values. ALB: albumin; ALT: alanine aminotransferase; CK: creatine kinase; DBP: diastolic blood pressure; FT4: free thyroxine; GGT: gamma-glutamyl transferase; GLU: glucose; INR: international normalized ratio; LDL: low-density lipoprotein cholesterol; PLT: platelet count; SBP: systolic blood pressure; UACR: urine albumin-to-creatinine ratio; UPCR: urine protein-to-creatinine ratio; WBC: white blood cell count.

For all-cause mortality, Figure 4A displays feature importance rankings from the XGBoost model. Hemoglobin and loop diuretic use were the most impactful features, followed by age, systolic blood pressure, and cancer. Figure 4B shows the SHAP summary plot for all-cause mortality. Lower hemoglobin levels, loop diuretic use, older age, lower systolic blood pressure, and the presence of cancer were generally associated with a higher predicted mortality risk.

### Calibration and Decision-Curve Analysis

**Figure 5.**
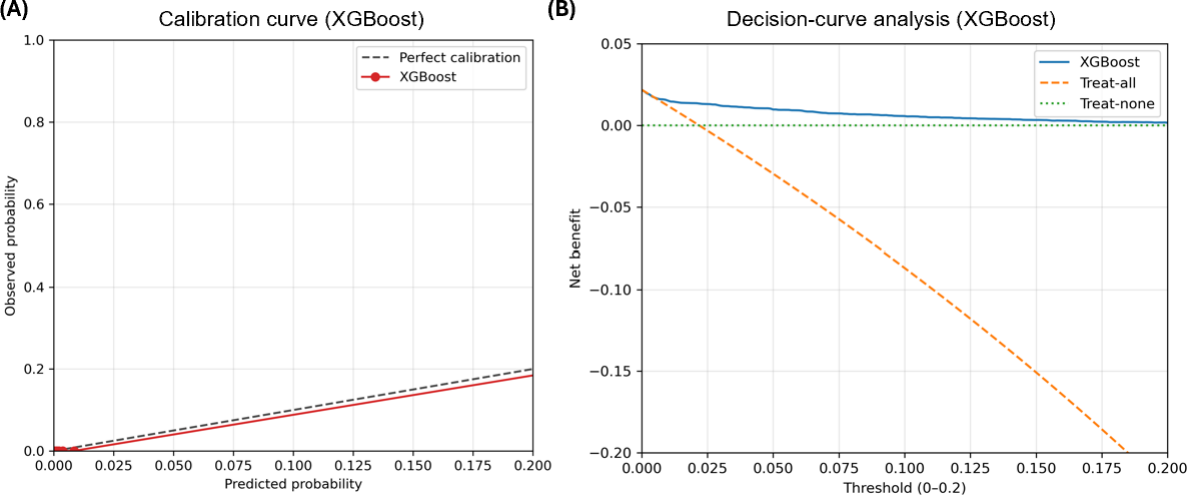
Calibration and decision-curve analyses of the extreme gradient boosting (XGBoost) model for end-stage renal disease prediction in the testing set. (A) Calibration curve comparing predicted and observed event probabilities. (B) Decision-curve analysis comparing the net benefit of the XGBoost model with the treat-all and treat-none strategies.

**Figure 6.**
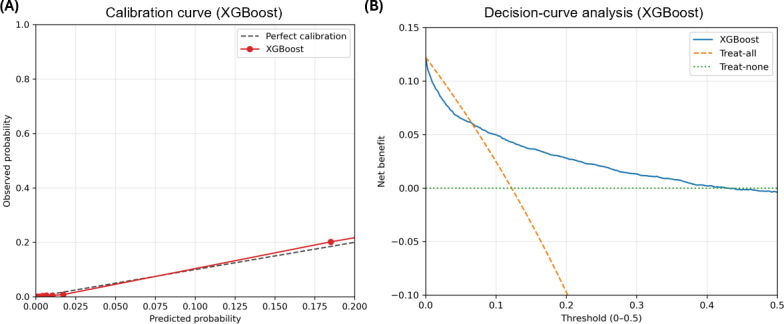
Calibration and decision-curve analyses of the extreme gradient boosting (XGBoost) model for all-cause mortality prediction in the testing set. (A) Calibration curve comparing predicted and observed event probabilities. (B) Decision-curve analysis comparing the net benefit of the XGBoost model with the treat-all and treat-none strategies.

To further assess clinical usefulness, calibration and decision-curve analyses of the XGBoost model were performed in the testing set. The calibration curve showed overall agreement between predicted and observed ESRD risks (Figure 5A). In the decision-curve analysis, the XGBoost model showed a higher net benefit than the treat-all and treat-none strategies across threshold probabilities ranging from 0 to 0.20 (Figure 5B). For all-cause mortality, the calibration curve also showed overall agreement between predicted and observed risks (Figure 6A). In the decision-curve analysis, the XGBoost model showed a positive net benefit across a broad range of threshold probabilities, although the treat-all strategy provided slightly higher net benefit at very low thresholds (Figure 6B).

### Survival Analysis Using RSF and Comparison With KFRE

**Figure 7.**
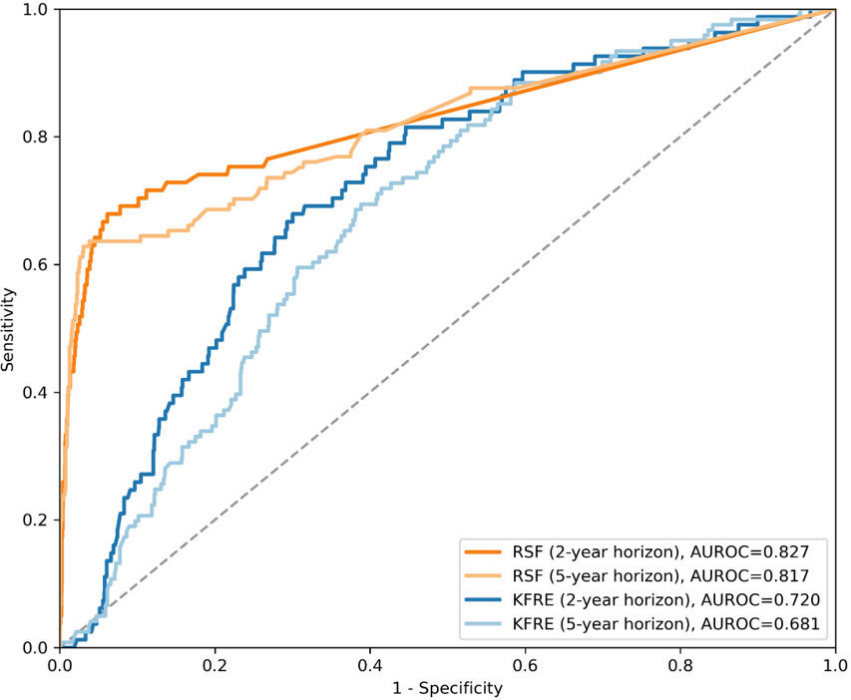
Receiver operating characteristic curves comparing the random survival forest (RSF) and the kidney failure risk equation (KFRE) for the prediction of end-stage renal disease at the 2- and 5-year horizons. AUROC: area under the receiver operating characteristic curve.

To better account for the time-to-event nature of ESRD, we additionally performed survival analysis using RSF. The predictive performance of RSF was then compared with that of the KFRE at the 2- and 5-year horizons (Figure 7). RSF demonstrated better discrimination than KFRE at both time points, with AUROCs of 0.827 versus 0.720 at 2 years and 0.817 versus 0.681 at 5 years.

### Sensitivity Analyses

In the age-stratified analysis, model discrimination remained similar between patients aged ≥60 years and <60 years, with AUROCs of 0.896 (95% CI 0.867‐0.921) and 0.866 (95% CI 0.777‐0.939), respectively (Figure S2 in Multimedia Appendix 1). In analyses without imputation, AUROCs for ESRD ranged from 0.888 to 0.903 and AUROCs for all-cause mortality ranged from 0.797 to 0.808 across models (Figure S3 in Multimedia Appendix 1), showing results comparable to the primary analysis.

## Discussion

In this study, we developed and validated machine learning models to predict the risk of ESRD and all-cause mortality in a large cohort of patients with CKD. Our findings showed that the evaluated machine learning algorithms, including XGBoost, LightGBM, CatBoost, random forest, and a stacking classifier, achieved good discrimination for ESRD prediction, with a maximum AUROC of 0.894. Predictive performance for all-cause mortality was more modest, with a maximum AUROC of 0.774. In addition, calibration and decision-curve analyses supported the overall reliability and clinical utility of the model. A previous study developed predictive models for CKD progression using traditional statistical methods and clinical variables from Canadian cohorts collected between 2001 and 2008 [7]. These models, based on Cox proportional hazards regression, showed reasonable predictive performance by incorporating key demographic and laboratory parameters, such as eGFR and albuminuria. However, their reliance on conventional statistical techniques and a limited number of variables may restrict their ability to fully capture complex relationships among CKD progression risk factors. Furthermore, evolving clinical practices, therapeutic strategies, and diagnostic methods over time might limit their applicability to contemporary patient populations.

Using advanced machine learning techniques allows the integration of a broader range of clinical, demographic, and laboratory features into predictive models, enabling more precise risk assessment for CKD progression [16,17]. Unlike traditional statistical methods, which are often constrained by linear assumptions and limited predictors, machine learning algorithms can capture complex, nonlinear interactions among variables. Additionally, these models manage high-dimensional data, autonomously identify relevant features, and continuously adapt to evolving clinical practices and patient populations. The incorporation of explainability tools, such as SHAP, further enhances interpretability by clarifying individual risk factor contributions [18]. A recent study by Tangri et al [19] validated the Klinrisk machine learning model using data from the CANVAS (Canagliflozin Cardiovascular Assessment Study) and CREDENCE (Canagliflozin and Renal Events in Diabetes With Established Nephropathy Clinical Evaluation) clinical trials, demonstrating superior performance compared to KDIGO (Kidney Disease: Improving Global Outcomes) risk classification, with an AUROC of 0.88 at 3 years. In addition, our survival-based comparative analysis showed that RSF outperformed the KFRE at both the 2- and 5-year horizons, suggesting that machine learning approaches incorporating time-to-event information may provide improved discrimination over conventional risk equations.

Another study used machine learning models to predict ESRD risk in newly diagnosed patients with type 2 diabetes mellitus (T2DM), illustrating the effectiveness of these approaches in risk stratification [10]. Among the evaluated algorithms, XGBoost achieved the highest predictive performance. This study also used SHAP to interpret predictions, identifying baseline serum creatinine, mean serum creatinine within 1 year before T2DM diagnosis, high-sensitivity C-reactive protein, and UPCR as key predictors. However, its limitation to patients with diabetes restricts generalizability to broader CKD populations. Given CKD’s heterogeneity, with significantly varying etiologies and progression rates between patients with diabetes and those without diabetes, models trained exclusively on T2DM populations might not fully capture CKD’s complexity. Our study extends the application of machine learning in CKD risk prediction by analyzing CKD cohort with varied etiologies, offering a comprehensive ESRD risk assessment beyond diabetes-related kidney disease. Previous machine learning studies predicting CKD progression often had limited sample sizes and narrow feature sets [20-22]. Our study, in contrast, uses a significantly larger cohort and broader clinical, laboratory, and demographic features, likely enhancing observed model performance. This comprehensive approach improves the applicability of machine learning–based models in clinical settings, aiding early identification and intervention for patients at high risk of ESRD.

We identified baseline serum creatinine, UACR, and UPCR as top predictors of ESRD, consistent with established CKD progression knowledge. These biomarkers have long been recognized as key indicators of kidney function decline, and their prominence reinforces their critical role in risk assessment. Hemoglobin also emerged as a strong predictor, suggesting anemia’s potentially significant role in CKD progression. Anemia is common in CKD, primarily due to reduced erythropoietin production, iron deficiency, and chronic inflammation []. It has been linked to increased cardiovascular risk and impaired quality of life, particularly among patients with CKD [23-25]. A study using data from the SOLVD trial, enrolling patients with heart failure with significantly reduced left ventricular ejection fractions of 35% or less, showed anemia significantly associated with accelerated eGFR decline, especially pronounced in patients with baseline CKD [26]. The complex interaction among anemia, cardiovascular disease, and renal function decline underscores the importance of comprehensive clinical management in CKD [27].

The predictive performance for all-cause mortality was lower than that for ESRD, likely reflecting the greater heterogeneity of mortality as an outcome. Unlike ESRD, which is more directly linked to renal function and proteinuria-related measures, all-cause mortality is influenced by a broader range of renal and nonrenal factors. Routinely collected structured EHR variables may not fully capture important dimensions of mortality risk, such as frailty, functional status, nutritional reserve, cognitive impairment, and overall disease severity. Incorporating these variables may improve mortality prediction in future studies. Top predictors identified by SHAP analysis were hemoglobin, loop diuretic use, age, systolic blood pressure, cancer, and albumin. In patients with CKD, lower hemoglobin levels may reflect reduced erythropoietin production, chronic inflammation, malnutrition, and a greater comorbidity burden, all of which can contribute to vulnerability and adverse outcomes [28]. Loop diuretic use may indicate underlying volume overload or more advanced heart or kidney disease. Lower systolic blood pressure also emerged as an important predictor of mortality. This finding is consistent with prior studies in older adults with CKD and may be especially relevant in our cohort, given its advanced age distribution [29]. In this setting, lower systolic blood pressure may reflect frailty, impaired cardiac function, malnutrition, or overall physiologic vulnerability. Serum albumin is a well-established marker of both nutritional status and systemic inflammation [30,31]. Hypoalbuminemia has been consistently linked to increased short- and long-term mortality across diverse patient populations, including those with and without chronic kidney disease [32-34]. Proteinuria, commonly used to assess renal progression, also reflects systemic endothelial dysfunction and microvascular damage, contributing to increased cardiovascular morbidity and mortality. Studies have linked proteinuria with increased risk of coronary artery disease, stroke, heart failure, and arrhythmias [35-39]. Even low-grade albuminuria has been associated with adverse cardiac remodeling and unfavorable cardiovascular outcomes. A recent large-scale cohort study further demonstrated that proteinuria significantly increases the risk of sudden cardiac arrest in young adults, particularly in those with CKD [40]. Despite its well-established prognostic value in cardiovascular disease, proteinuria remains underutilized in routine cardiovascular risk stratification [41]. Our findings reinforce the clinical importance of proteinuria and support its broader integration into multidisciplinary risk evaluation strategies, especially within CKD populations.

From a clinical perspective, the practical value of these machine learning models lies in their potential integration into routine electronic health record workflows. Such risk estimates may help clinicians identify patients at high risk for ESRD or death who may benefit from closer follow-up, earlier nephrology referral, intensified management of modifiable risk factors, and more timely preparation for kidney replacement therapy. In addition, the decision-curve analysis suggests that the model may support risk-based clinical decision-making across a range of threshold probabilities. However, the optimal thresholds for intervention and the clinical impact of implementing such a model should be established in future prospective studies.

The strengths of our study include a large real-world CKD dataset with comprehensive clinical and laboratory information. Multiple machine learning algorithms, enhanced by advanced techniques such as SMOTE and SHAP, strengthen our findings’ robustness and applicability. Consistency in model performance across different frameworks further supports reliability. However, several limitations should be acknowledged. First, this was a retrospective, single-center study, which may introduce selection bias and limit generalizability. In particular, the cohort had a relatively advanced age distribution, likely reflecting the older patient population served by our institution. Nevertheless, age-stratified subgroup analysis showed similar model discrimination in patients aged ≥60 and <60 years, although external validation in younger populations remains necessary. Second, although we included a broad set of routinely available clinical variables, some potentially relevant factors, such as genetic markers and socioeconomic influences, were not included because they were not consistently available in structured form in our EHRs. Future studies incorporating multiomics data and social determinants may enhance predictive accuracy. Third, preprocessing methods may have introduced bias. KNN imputation may smooth extreme laboratory values, and SMOTE may generate synthetic minority samples that do not fully reflect the true clinical distribution, particularly for laboratory-based predictors. However, sensitivity analyses without imputation showed comparable model discrimination, suggesting that the overall results were broadly similar without imputation. Finally, the relative homogeneity of this hospital-based cohort may limit broader generalizability, and external validation in independent and more diverse CKD populations remains necessary.

In conclusion, machine learning models may provide useful tools for risk stratification of ESRD and all-cause mortality in patients with CKD. However, further validation is required before broader clinical application.

## Supplementary material

10.2196/81152 Multimedia Appendix 1 Model performance metrics, confusion matrices, age-stratified receiver operating characteristic curves, and sensitivity analyses without imputation for the prediction of end-stage renal disease and all-cause mortality.

## Abbreviations

AUPRC: area under the precision-recall curve
AUROC: area under the receiver operating characteristic curve
CANVAS: Canagliflozin Cardiovascular Assessment Study
CatBoost: categorical boosting
CKD: chronic kidney disease
CREDENCE: Canagliflozin and Renal Events in Diabetes With Established Nephropathy Clinical Evaluation
eGFR: estimated glomerular filtration rate
EHR: electronic health record
ESRD: end-stage renal disease
KDIGO: Kidney Disease: Improving Global Outcomes
KFRE: kidney failure risk equation
LightGBM: light gradient boosting machine
RSF: random survival forest
SHAP: Shapley additive explanations
SMOTE: Synthetic Minority Over-Sampling Technique
T2DM: type 2 diabetes mellitus
UACR: urine albumin-to-creatinine ratio
UPCR: urine protein-to-creatinine ratio
XGBoost: extreme gradient boosting
