# Supplementary material for: Predicting End-Stage Renal Disease and Mortality in Chronic Kidney Disease Using Machine Learning: Retrospective Cohort Study
Source: JMIR Med Inform. 2026 Jun 5;14:e81152. doi: 10.2196/81152 (PMC13240987; doi:10.2196/81152)

**Predicting End-Stage Renal Disease and Mortality in Chronic Kidney Disease Using Machine Learning: A Retrospective Cohort Study**

**Authors:** Tz-Heng Chen, MD, Kuan-Hsun Lin, Yang Ho, MD, Wei-Cheng Tseng, MD, PhD, Yuan-Chia Chu, PhD, Der-Cherng Tarng, MD, PhD

**Table of Contents**

| **Supplementary Tables** |  |
| --- | --- |
| Supplementary Table S1. The performance of machine learning models after data augmentation for end-stage renal disease prediction | 2 |
| Supplementary Table S2. The performance of machine learning models for all-cause mortality prediction | 2 |
| **Supplementary Figures** |  |
| Supplementary Figure S1. Confusion matrices of the XGBoost model for prediction of (A) end-stage renal disease and (B) all-cause mortality in the testing set. | 3 |
| Supplementary Figure S2. Receiver operating characteristic curves of the XGBoost model for end-stage renal disease prediction, stratified by age group (<60 years vs ≥60 years). | 4 |
| Supplementary Figure S3. Receiver operating characteristic curves of the machine learning models for prediction of (A) end-stage renal disease and (B) all-cause mortality without imputation. | 5 |

**Supplementary Tables**

**Supplementary Table S1. The performance of machine learning models after data augmentation for predicting the risk of end-stage renal disease**

|  | **Accuracy** | **Precision** | **Recall** | **F1 Score** | **AUC** | **Specificity** | **Loss** |
| --- | --- | --- | --- | --- | --- | --- | --- |
| XGBoost | 0.937 | 0.180 | 0.531 | 0.268 | 0.877 | 0.946 | 0.091 |
| LightGBM | 0.949 | 0.210 | 0.485 | 0.293 | 0.875 | 0.959 | 0.087 |
| CatBoost | 0.935 | 0.170 | 0.508 | 0.254 | 0.856 | 0.944 | 0.096 |
| Random Forest | 0.836 | 0.098 | 0.785 | 0.174 | 0.882 | 0.838 | 0.292 |
| Stacking Classifier | 0.943 | 0.188 | 0.485 | 0.270 | 0.885 | 0.953 | 0.116 |

Abbreviations: AUC, areas under the receiver operating characteristic curve; CatBoost, categorical boosting; XGBoost, eXtreme Gradient Boosting; LightGBM, light gradient boosting machine.

**Supplementary Table S2. The performance of machine learning models for all-cause mortality prediction**

|  | **Accuracy** | **Precision** | **Recall** | **F1 Score** | **AUC** | **Specificity** | **Loss** |
| --- | --- | --- | --- | --- | --- | --- | --- |
| XGBoost | 0.757 | 0.279 | 0.623 | 0.385 | 0.770 | 0.757 | 0.279 |
| LightGBM | 0.808 | 0.313 | 0.479 | 0.379 | 0.758 | 0.808 | 0.313 |
| CatBoost | 0.825 | 0.340 | 0.461 | 0.392 | 0.758 | 0.825 | 0.340 |
| Random Forest | 0.708 | 0.250 | 0.694 | 0.368 | 0.768 | 0.708 | 0.250 |
| Stacking Classifier | 0.823 | 0.338 | 0.466 | 0.391 | 0.769 | 0.823 | 0.338 |

Abbreviations: AUC, areas under the receiver operating characteristic curve; CatBoost, categorical boosting; XGBoost, eXtreme Gradient Boosting; LightGBM, light gradient boosting machine.

**Supplementary Figure S1.** Confusion matrices of the XGBoost model for prediction of (A) end-stage renal disease and (B) all-cause mortality in the testing set.


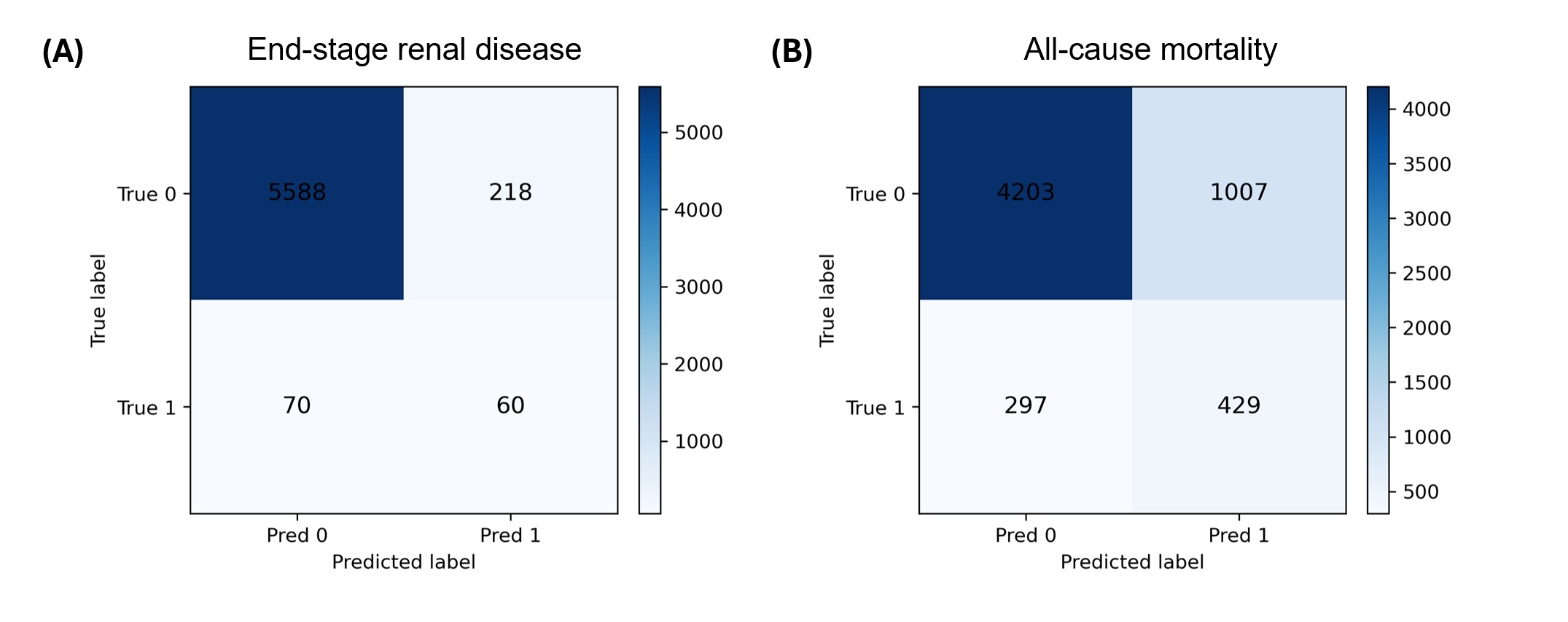


**Supplementary Figure S2.** Receiver operating characteristic curves of the XGBoost model for end-stage renal disease prediction, stratified by age group (<60 years versus ≥60 years). Abbreviations: AUROC, area under the receiver operating characteristic curve; CI, confidence interval; XGBoost, eXtreme Gradient Boosting.


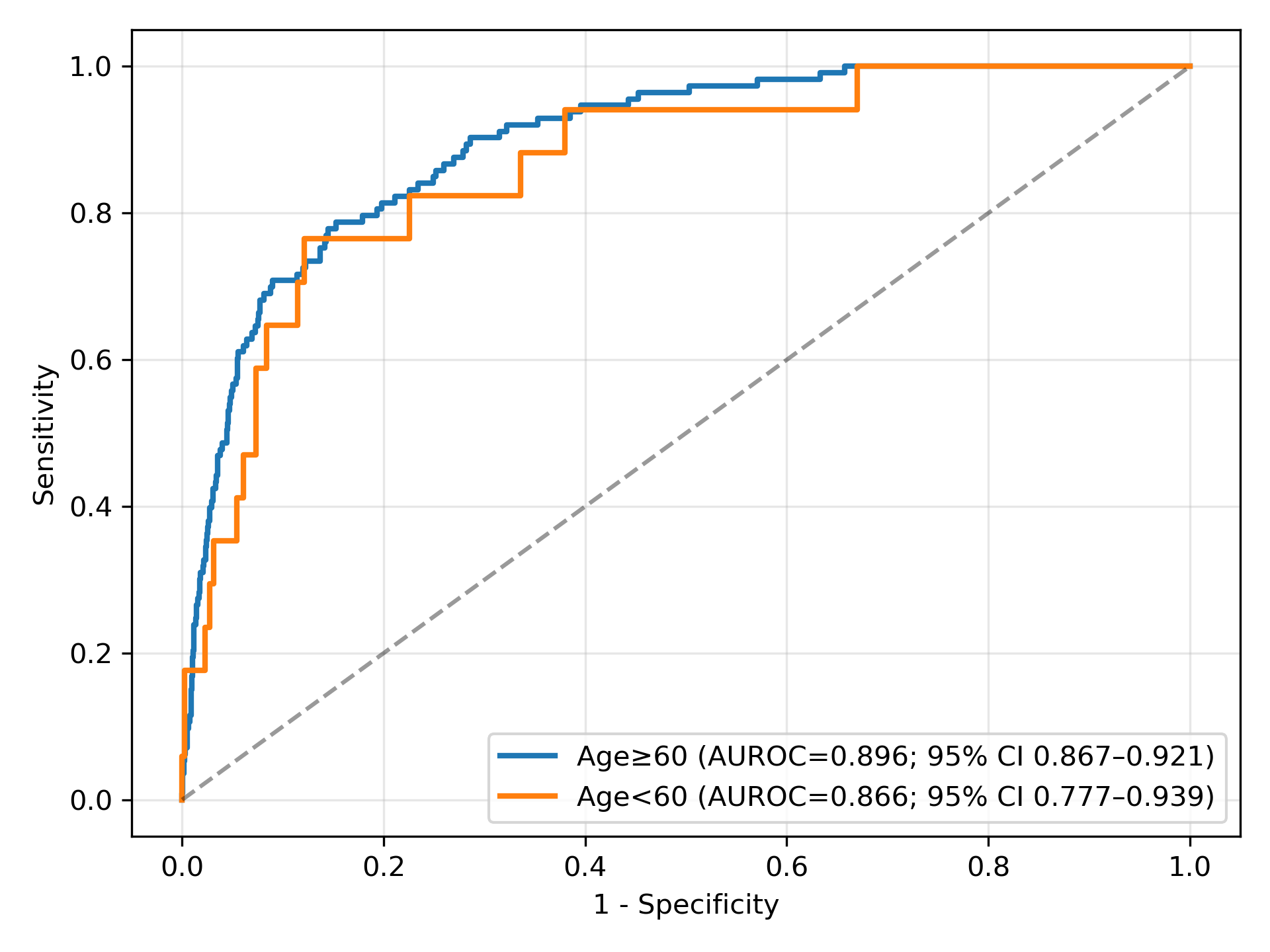


**Supplementary Figure S3.** Receiver operating characteristic curves of the machine learning models for prediction of (A) end-stage renal disease and (B) all-cause mortality without imputation. Abbreviations: AUROC, area under the receiver operating characteristic curve; XGBoost, eXtreme Gradient Boosting; LightGBM, light gradient boosting machine; CatBoost, categorical boosting.


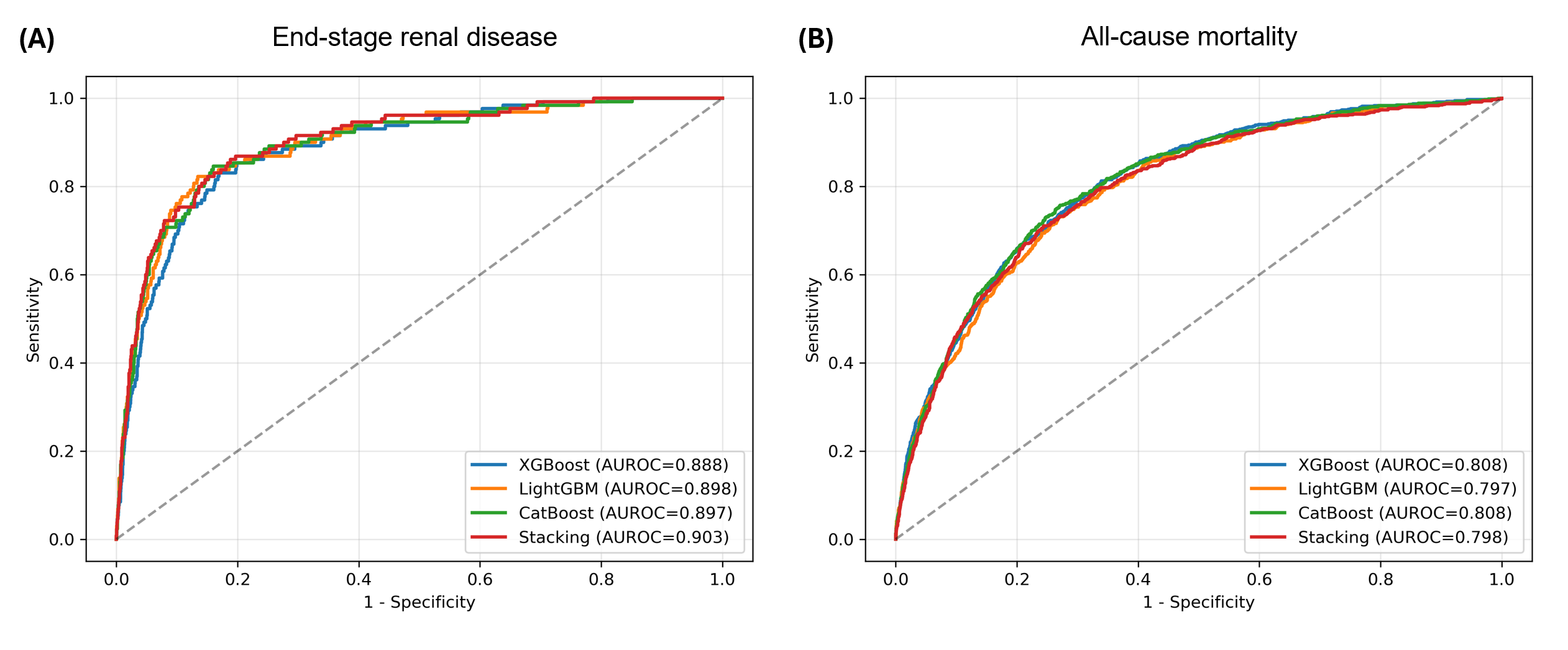

Supplement: Multimedia Appendix 1 [file medinform-v14-e81152-s001.docx]
